# Development of Phantoms for Multimodal Magnetic Resonance Imaging and Magnetic Particle Imaging

**DOI:** 10.3390/polym14193925

**Published:** 2022-09-20

**Authors:** Maria Alejandra Ardila Arenas, Dirk Gutkelch, Olaf Kosch, Rüdiger Brühl, Frank Wiekhorst, Norbert Löwa

**Affiliations:** 1 Working Group 8.23 Metrology for Magnetic Nanoparticles, Physikalisch-Technische Bundesanstalt, 10587 Berlin, Germany; 2 Departamento de Ciencias Aplicadas, Institución Universitaria ITM, Medellin 050034, Colombia; 3 B-Smart Lab, Physikalisch-Technische Bundesanstalt, 10587 Berlin, Germany; 4 Working Group 8.12 In Vivo MRI, Physikalisch-Technische Bundesanstalt, 10587 Berlin, Germany

**Keywords:** additive manufacturing, phantoms, magnetic nanoparticles, biomedical imaging, multimodal imaging, photopolymers

## Abstract

Phantoms are crucial for the development of imaging techniques based on magnetic nanoparticles (MNP). They serve as test objects to simulate application scenarios but are also used for quality assurance and interlaboratory comparisons. Magnetic particle imaging (MPI) is excellent for specifically detecting magnetic nanoparticles (MNP) without any background signals. To obtain information about the surrounding soft tissue, MPI is often used in combination with magnetic resonance imaging (MRI). For such application scenarios, this poses a challenge for phantom fabrication, as they need to accommodate MNP as well as provide MR visibility. Recently, layer-by-layer fabrication of parts using Additive Manufacturing (AM) has emerged as a powerful tool for creating complex and patient-specific phantoms, but these are characterized by poor MR visibility of the AM material. We present the systematic screening of AM materials as candidates for multimodal MRI/MPI imaging. Of all investigated materials, silicone (Dreve, Biotec) exhibited the best properties with sufficient MR-signal performance and the lowest absorption of MNP at the interface of AM materials. With the help of AM and the selection of appropriate materials, we have been able to produce suitable MRI/MPI phantoms.

## 1. Introduction

Magnetic particle imaging (MPI) is an emerging quantitative imaging technology visualizing the 3D distribution of magnetic nanoparticles (MNP) used as MPI tracers, in vivo [1]. However, since the signal is generated only by the MPI tracer, the surrounding biological tissue cannot be imaged directly. Therefore, a complementary imaging technique, such as magnetic resonance imaging (MRI) or computer tomography (CT), is necessary to provide anatomical information. Since MRI is the imaging technique with the best soft tissue contrast, MRI is often used as a complementary method in preclinical MPI imaging experiments [2,3,4]. Typically, the anatomy (or soft tissue contrast) of the subject is first recorded in an MRI device. Then the subject is brought into the MPI scanner for detecting the MNP distribution. An image fusion technique is used to integrate complementary information into one image.

Phantoms are essential for testing hardware, reconstruction algorithms, co-registration procedures, and other imaging performance metrics. Commercially available phantoms may be sufficient for daily routine checks or calibration, but they are unsuitable for specific scenarios, such as simulations of in vivo experiments. In contrast, Additive Manufacturing (AM) can be used to produce customized phantoms for specific research tasks. Even anatomical replicas of a particular research subject can be realized using AM. In preclinical research, these customized models can be used to train new and challenging use cases. Cost-effective on-site production is particularly advantageous, especially for single-use experiments to test new ideas. The basic prerequisite for this is the availability of equipment and suitable materials. Among available AM processes, vat photopolymerization is usually considered the technology that can achieve the highest standards in terms of accuracy, resolution, and repeatability [5]. Vat photopolymerization is an AM process in which a liquid photopolymer (liquid monomers, oligomers, and photoinitiators) is selectively cured layer by layer in a vat using a light source. In this reaction, a photoinitiator molecule in the photopolymer is triggered by the incident light and locally activates a chemical polymerization reaction that leads to curing only in the exposed areas. There are several variations of vat photopolymerization, the best known of which are stereolithography (SLA) and digital light processing (DLP).

In MPI imaging, it is advantageous if the phantoms serve both imaging modalities, MPI and MRI, simultaneously. MPI phantoms are usually solid bodies with cavities of different sizes and geometries filled with different concentrations of MPI tracer [6,7,8]. Biomimetic phantoms with cavities corresponding to anatomical geometries are used to simulate the uptake of MPI tracer in a given organ [9]. Some studies have used AM methods to generate physiological structures filled with MNP for MPI but have not investigated their suitability for MRI [9,10,11]. In addition, special consideration should be given to the interaction of phantom materials with the MPI tracer, since nonspecific absorption on the material surface [12,13] may lead to signal alterations of the immobilized tracer [14]. Furthermore, it is important to ensure that these materials do not produce magnetic signals [15].

MRI phantoms, on the other hand, are usually made of a gel (e.g., agar) to achieve the desired imaging properties of human tissue. These types of phantoms are usually produced by conventional processing techniques, such as molding and casting. Research on MR visible AM materials is of great interest and the first successful material developments have already been reported [16,17,18]. The suitability of such AM materials for magnetic particle imaging has not yet been reported. In these existing forms, neither MPI nor MRI phantoms are compatible for imaging with the other modality. 

In this work, we present the development of a multimodal MRI/MPI-phantom using DLP-based AM. We address the challenges in selecting appropriate AM materials for the fabrication of MPI/MRI phantoms. Physical parameters of five commercially available materials for AM were evaluated, including absorption (of MPI-tracer), magnetic background signal (contamination), MRI performance, shore hardness, and aging. Finally, we tested a dedicated MPI/MRI phantom made of appropriate material in both imaging modalities. 

## 2. Materials and Methods

### 2.1. Materials

Different liquid photoreactive acrylic photopolymers for digital light processing (DLP) based AM (i30+, Rapidshape, Heimsheim, Germany) were used in this work. E-Shell 200 and E-Shell 600 (Envisiontec GmbH, Gladbeck, Germany) are low viscosity liquid photopolymers. They can be used to produce strong, tough, water-resistant ABS-like parts with high detail. Both materials are CE-certified and biocompatible according to ISO 10993 class IIa. The photoreactive resin E-Shell 600 contains 60–80% acrylic monomer, 10–25% tetrahydrofurfuryl methacrylate, 5–20% urethane dimethacrylate and <1% diphenyl(2,4,6-trimethylbenzoyl)phosphine oxide [19,20] whereas E-Shell 200 contains 60–80% 7,7,9-Trimethyl-4,13-dioxo-3,14-dioxa-5,12-diaza-hexadecan-1,16-diol dimethacrylate, 20–40% Neopentyl glycol propoxylate diacrylate, <3% phosphine oxide [21].

R5 Gray and R5 Red (Envisiontec GmbH, Gladbeck, Germany) are colored photopolymers that feature high durability, good stiffness, high surface quality, excellent fatigue resistance, chemical resistance, and excellent tolerance to a wide range of temperatures and humidity. The R5 series contains 20–60% acrylated oligomer, 5–30% acrylated monomer, 10–25% isobornyl acrylate, and 1–2% phosphine oxide [22].

BioTec (Dreve, Unna, Germany) is a directly printable, highly elastic silicone material to produce soft parts, which are characterized by their tear resistance, elasticity, and high surface quality. The final hardness is 50 55 Shore A. BioTec contains 10–25% 3,3,5-trimethylcyclohexylacrylate, 2.5–10% dimethylethylcyclohexylacrylate, 2.5–10% 2-ethylhexyl acrylate, and <2.5% diphenyl(2,4,6-trimethylbenzoyl)phosphinoxide [23]. Similar properties are offered by Elastic 50A (Formlabs, Somerville, MA, USA), which is used to produce parts that simulate silicone. This material was the only one processed on a stereolithography (SLA) based AM system (Form 2, Formlabs, Somerville, MA, USA). Elastic 50A contains 15–25% isobornyl acrylate, and 25–45% acrylate monomers [24].

COOH coated MNP of type Synomag^®^ (Micromod Partikeltechnologie GmbH, Rostock, Germany) with a hydrodynamic size of *d*_h_ = 49.7 nm and a negative zeta-potential of *ξ* = −35 mV was used for MPS and MPI experiments.

### 2.2. In-Process and Post-Process Procedure

A series of test specimens was prepared using AM. For each specimen, five replicates were manufactured in the same batch. Presented results are means of these five replicates. For magnetic characterization, cylindrical reference samples (height 5.3 mm, diameter 5.3 mm) were manufactured. Hardness tests were conducted on cuboids (20 × 20 mm, height 6 mm). A cylindrical phantom (height 75 mm, diameter 52 mm) for testing MRI/MPI imaging properties with cavities of different diameters (1, 1.5, 2, 2.5, 3, 4 mm) arranged parallel in the longitudinal axis of the cylinder was finally fabricated by DLP-based AM only.

The post-cure was carried out to achieve complete polymerization, improve the layer adhesion, and reduce the anisotropic properties of the parts. SLA parts were post-cured using the Form Cure (Formlabs, Somerville, MA, USA) at 60 °C for 20 min. DLP parts were treated in the UVA-Cube 2000 (Dr. Hönle AG, Graefelfing, Germany) for 8 min at 1000 W.

### 2.3. Examination Methods

#### 2.3.1. Shore A Hardness Test

The printed parts were examined with a shore A hardness tester (SAUTER HDA 100-1, Kern & Sohn GmbH, Balingen, Germany). The hardness of the outer surfaces of each sample was determined as an average value after five measurements from one side. All measurements were performed according to DIN ISO 7619-1.

#### 2.3.2. Magnetic Particle Spectroscopy (MPS)

The materials were checked for possible magnetic impurities and unwanted MPI tracer absorption on the AM material by MPS (i.e., 0-dimensional MPI) using a commercial device (MPS-03, Bruker BioSpin, Ettlingen, Germany). 

During a measurement, the sample is exposed to a sinusoidally oscillating magnetic field (excitation amplitude of *B* = 25 mT, frequency *f*_0_ of 25 kHz) and the nonlinear magnetization response is detected (sensitivity: 5 pAm^2^). The fundamental excitation frequency *f*_0_ is strongly suppressed by filtering and gradiometric design of the receive coil. Fourier transformation of the time signal yields the characteristic MPS amplitude spectrum *A*_n_ with pronounced odd multiples (n = 3, 5, 7, etc.) of the excitation frequency *f*_0_ (n = 1). The amplitudes *A*_n_ of the MPS spectrum are directly proportional to the number of MNP (or amount of iron) used and decrease monotonically with the harmonic number n. Therefore, the strongest amplitude *A*_3_ is mainly used for quantification. 

For the quantification of MNP iron in a sample, the signal amplitude *A*_3_ of a reference sample with known iron content was first determined. The unknown iron content of a sample can be determined from the reference sample signal (of the same MNP type) based on the measured signal amplitude *A*_3_.

#### 2.3.3. Time-Domain Nuclear Magnetic Resonance (TD-NMR)

TD-NMR was used to determine the MRI performance of the samples. The transverse relaxation time *T*_2_ was measured, using the CPMG pulse sequence on a low-field NMR analyzer (minispec mq60) from Bruker BioSpin (Ettlingen, Germany). 

#### 2.3.4. Magnetic Particle Imaging (MPI)

To analyze the MPI imaging performance of the final cavity phantom, a commercial preclinical MPI scanner (MPI 25/20 FF, Bruker BioSpin, Ettlingen, Germany) was used. This system is a field-free point scanner using the system function approach for image reconstruction. Field gradients of 1.5 T/m in z- and 0.75 T/m in x and y and a drive field amplitude of 12 mT in all three directions were used for spatial encoding. The Kaczmarz-Algorithm was applied with 20 iterations and a regularization factor of *λ*_r_ = 10^−5^ for image reconstruction.

#### 2.3.5. Magnetic Resonance Imaging (MRI)

The MR scan was performed on a 3T Verio system (Siemens AG, Erlangen, Germany) using a 15-channel Tx/Rx knee coil. A 3D-FLASH sequence (with TE = 3.2 ms, TR = 620 ms, 128 × 128 × 80 matrix, 0.78 × 0.78 × 1.1 mm^3^ resolution, 32 repetitions) acquired the image in 25 min. Chemical shift artefacts occur in the frequency-encoded direction from left to right.

## 3. Results and Discussion

### 3.1. Material Selection

We evaluated different AM materials for their suitability as phantoms for MNP-based imaging. 

**Figure 1:**
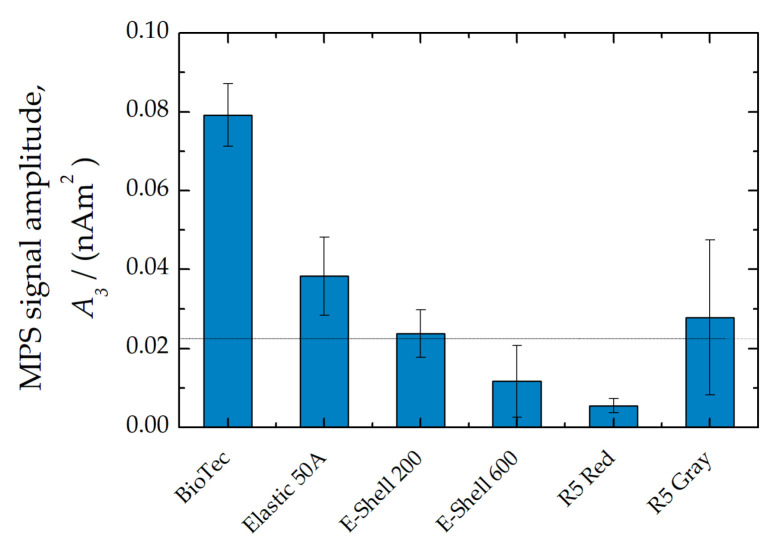
MPS signal of raw sample materials. The horizontal line denotes the threefold standard deviation of 100 blank measurements and defines the apparent limit of detection (LOD).

First, it was investigated whether the materials already cause a magnetic signal in their initial state. For this purpose, cylindrical test bodies were analyzed using MPS. It was found that the signal amplitude *A*_3_ of the flexible materials BioTec and Elastic 50A were significantly above the limit of detection limit (LOD) of the MPS device (Figure 1). The signal amplitude *A*_3_ of the other materials was close to or below the LOD, indicating low magnetic contamination. 

For this reason, the liquid photopolymers were also examined in their initial state with MPS. Here, the signals from BioTec and Elastic 50A were below the LOD. However, in contrast to the other materials, the surfaces of the samples made of the BioTec and Elastic 50A materials were very sticky. Therefore, it is assumed that the measured magnetic impurity was caused by the adhesion of magnetic dust. Dust in the ambient air is one of the main sources of magnetic contamination for the highly sensitive MPS instrument. Similar results were previously obtained when different tubing materials were examined, with silicone tubing showing the strongest contamination [25].

**Figure 2:**
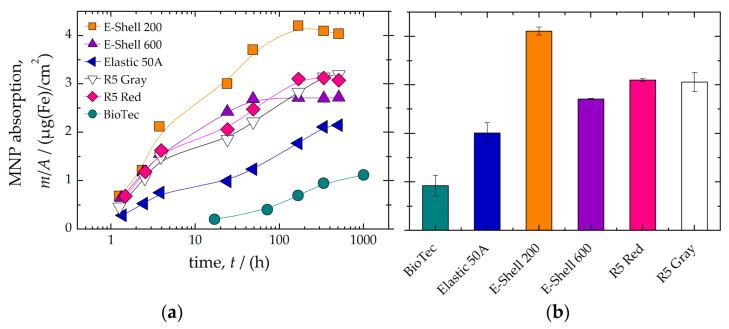
The time-dependent accumulation of MNP on the sample surface (shape cylinder) of different AM materials was quantified by MPS (**a**). Saturation value of absorbed MNP (MPI tracer) amount after 300 h for each AM material (**b**).

Furthermore, we investigated whether prolonged exposure to MPI tracer leads to absorption by the AM materials. Therefore, cylindrical test bodies of each AM material were immersed in a suspension containing MPI tracer (*c*(Fe) = 10 mmol/L). At specific time points (1 min to 6 weeks), the samples were removed, washed three times in demineralized water, and the remaining MNP iron mass absorbed by the AM material was quantified using MPS (see Figure 2a). After about 300 h, it was found that all materials showed saturation of the absorbed MPI tracer. In particular, the AM materials BioTec and Elastic 50A showed the lowest absorption after an incubation time of 300 h (Figure 2b). The silicone material is known to develop static electricity due to friction [26]. Therefore, the negative charge of the silicone material might have prevented the adhesion of the negatively charged MNP.

**Figure 3:**
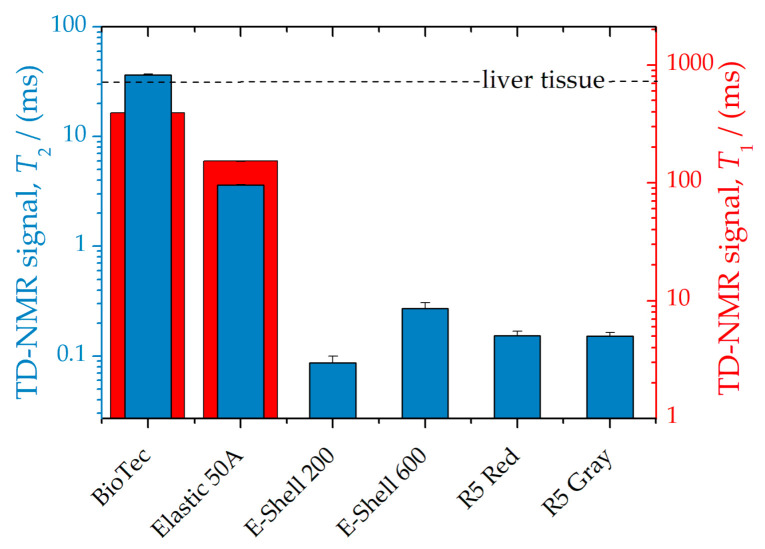
TD-NMR relaxation times *T*_1_ (red) and *T*_2_ (blue) were measured by TD-NMR of the cylindrical samples. The horizontal line denotes the *T*_2_ = 31 ms and *T*_1_ = 745 ms of liver tissue [27]. For *T*_2_ determination of BioTec and Elastic 50A samples, a double-exponential fit function was applied and the slower relaxation time was plotted as found previously by [28]. *T*_1_ relaxation values for E-Shell and R5 samples were not measurable with the TD-NMR relaxometer.

To investigate the suitability of the materials for MRI they were measured using TD-NMR. This revealed that only the BioTec material exhibited a *T*_2_ relaxation time close to that of soft tissue (liver, Figure 3). Interestingly, the exponential decay of the signal for both BioTec as well as Elastic 50A showed a fast relaxation time component (*T*_2_ about 1 ms) in addition to the slow component of 36.6 ms and 4 ms, respectively. In contrast, the measured *T*_1_ values did not quite reach the signal of soft tissue or were simply not measurable at all (E-Shell and R5 samples).

### 3.2. Phantom Development

Based on the results from Section 3.1 (see Table 1), BioTec was selected as the material for the AM of an MRI/MI phantom. To test the suitability of the material for permanent usage in a phantom, time-dependent material changes (shore A hardness, *T*_2_ relaxation time) were investigated for different storage conditions (without precautions, in a container, in a water bath). The first measurements were performed directly after the AM post-process (about 2 h to 5 h after the AM process). This was followed by storage under different conditions. Finally, all measurement results were normalized to the first measured value.

**Figure 4:**
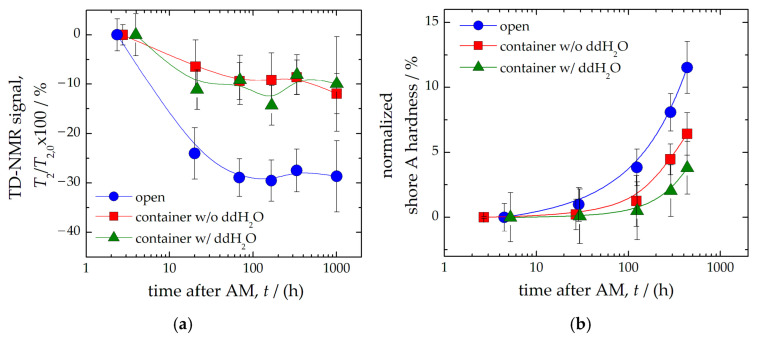
Measured transverse relaxation time *T*_2_ of samples at different storage conditions (**a**). Samples were stored without any precautions (blue circles), in a container (red squares), or in a container with water (green triangles). Shore A hardness was measured on similar samples (**b**). All signals were normalized to the initial values (*T*_2_ relaxation time of 36.6 ms, shore A hardness of 29). The solid lines are plotted as a guide to the eye.

It was found that normal storage without any precautions led to a significant change in the material properties (Figure 4). However, this could be due to the fact that polymerization reactions are rarely complete since not all bonds are completely converted. This leads to the presence of unreacted residual monomers in the polymer material (such as 3,3,5-trimethylcyclohexylacrylat in BioTec), which can cause late polymerization (i.e., post-hardening). In addition, a change in hardness may also be associated with the leaching of residual monomers and plasticizers, and the absorption of water [29,30,31,32,33]. Consequently, diffusion of residual (non-crosslinked) monomer into the solvent leads to weaker post-curing effects (i.e., lower hardness), while leaching of plasticizers (such as 2-ethylhexyl acrylate in BioTec) reduces sample hardness. In addition, diffusion of water into the material can lead to softening of the surface.

The measured *T*_2_ relaxation time in TD-NMR decreased by 30% after one day and remained constant at a low level thereafter (Figure 4a). In contrast, the signal loss when stored in a container or water was only 10%. Similar results were obtained by measuring the shore A hardness of the material (Figure 4b). The Shore A hardness of the samples tested without precautions increased faster (up to 10%) than when stored in a container or water. Thus, storing the phantom in a closed container (protected from drying out) or in a water bath permanently preserved the material and signal properties.

**Figure 5:**
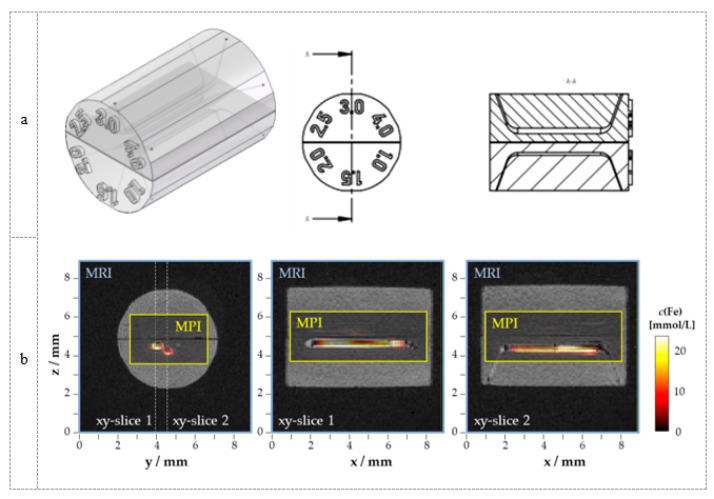
Phantom made of BioTec material (Dreve) for MPI and MRI imaging which contains capillaries with a diameter of 1, 1.5, 2, 2.5, 3, and 4 mm (**a**). MRI (blue border) and MPI (yellow border) results of the designed phantom with BioTec material are shown as image fusion (**b**). For MPI, a dedicated tracer (Synomag^®^, *c*(Fe) = 19.5 mmol/L) was filled into the 3 mm and 4 mm capillaries for imaging with MPI.

To demonstrate the capability for both MRI as well as MPI imaging, we manufactured a phantom made of BioTec material. This phantom was equipped with capillaries that could be filled with an MPI tracer for the MPI measurements (Figure 5a). MRI successfully visualized the phantom without MPI tracer in the capillaries. The unfilled capillaries were easily discriminated from the BioTec material by MRI (Figure 5b). After injection of MPI tracers into the capillaries (diameter 3 mm and 4 mm), they could also clearly be visualized by MPI (Figure 5b). The image fusion shows the geometrically correct representation of the tracer distribution using MPI in the capillaries. Due to the larger voxel size of MPI compared to the diameter of the capillaries, an exact determination of the capillary dimensions using MPI is not possible. However, with the help of higher gradients in MPI and tracers with superior MPI signals, this could become possible.

## 4. Conclusions

In this study, the potential of photopolymerization-based AM technology for the fabrication of multimodal MRI/MPI phantoms was investigated. The results show that a comprehensive characterization is necessary to select suitable AM materials for MRI/MPI-phantom fabrication. Ideally, the AM materials should not contain magnetic impurities, otherwise, the localization of MPI tracers using MPI will be affected by excessive background signals from the phantom. Furthermore, quantification of a non-specific absorption of the used MPI tracer by the phantom material is mandatory since the immobilization of these tracers leads to MPI signal alteration and disables the reuse of the phantom.

We found that the BioTec material showed the lowest absorption of MPI tracer. This is probably due to the negative charge of the silicone material, which prevents the adhesion of the negatively charged particles. Nevertheless, this AM material exhibited a significantly higher background signal, which was attributed to the sticky surface that makes magnetic dust adhesion more likely. Accordingly, phantoms made of this material should be particularly protected from environmental influences (e.g., magnetic dust, drying out). This also applies to the long-term stability of the MR signal. We found that already one day after AM fabrication, the MR signals of BioTec material decrease if the phantom is not stored in a closed container. Under these precautions, it is possible to produce phantoms from BioTec material for multimodal MRI/MPI imaging (e.g., liver phantoms). Finally, a phantom with capillaries of different diameters was developed to demonstrate multimodal MRI/MPI imaging on this phantom material. Using MRI, it was possible to image the phantom and associated fine structures (capillaries). After filling with a suitable MPI tracer, MPI imaging of the phantom was successfully demonstrated.

This approach will be applied in the future to produce phantoms that will be used for performance verification of multimodal MPI/MRI imaging. Furthermore, it will be investigated whether new material compositions can be used to generate MR signals similar to other soft tissues with longer relaxation times, such as white matter. Furthermore, it is possible to introduce MPI tracers directly into the still liquid photopolymers [8,15] and to process them afterwards by AM. Since the MPI tracers obliterate the MR signal, multi-material DLP printers must be used to produce compartments with and without MPI tracers [34].

## Figures and Tables

**Table 1:** Summary of the material selection procedure. A ranking is presented for each investigated property (I: best suitable, II: questionable suitability, III: unsuitable). The number in the parenthesis indicates the uncertainty in the last digit.

Material	Magnetic Impurity		MNP Absorption		TD-NMR Signal	
	*A*_3_	Ranking	*m/A*	Ranking	*T*_2_	Ranking
	pAm^2^		µg/m^2^		ms	
BioTec	79(8)	II	0.9(2)	I	36.6(5)	I
Elastic 50A	38(1)	I	2.0(2)	II	3.62(3)	III
E-Shell 200	23(6)	I	4.11(8)	III	0.09(1)	III
E-Shell 600	11(9)	I	2.71(1)	II	0.27(3)	III
R5 Red	6(2)	I	3.10(2)	II	0.15(1)	III
R5 Gray	28(2)	I	3.1(2)	II	0.15(1)	III
